# Recent Advances in Consumer Behavior Theory: Shocks from the COVID-19 Pandemic

**DOI:** 10.3390/bs11120171

**Published:** 2021-12-09

**Authors:** Bibo Yin, Yajing Yu, Xiaocang Xu

**Affiliations:** 1 College of Economic and Trade, Hunan University of Technology and Business, Changsha 410205, China; 2455@hutb.edu.cn; 2 College of Science, Hunan University of Technology and Business, Changsha 410205, China; 211020252042@stu.hutb.edu.cn; 3 School of Economics and Management, Huzhou University, Huzhou 313000, China

**Keywords:** COVID-19, consumer behavior, consumption willingness, consumption pattern

## Abstract

***Background:*** The COVID-19 pneumonia epidemic has had an enormous impact on people’s lives, particularly aspects of life such as consumption, and has therefore brought new elements to the expansion of Consumer behavior theory. ***Methods:*** This paper searches the literature on consumption research conducted from 1981 to 2021, including sources such as CNKI, Wanfang, Google Scholar, and Web of Science. Through the exploration of the existing relevant literature, this article found that the COVID-19 pneumonia epidemic has had a profound impact on consumption willingness, consumption patterns, and consumption objects, and, as such, has newly expanded the theoretical model of consumer behavior. ***Results:*** Through reviewing the literature, this paper found some results. For example with regard to consumption patterns, early studies and the impact of COVID-19 was focused on online consumption, however in the context of COVID-19, scholars proposed paying attention to the combination of online and offline development. ***Conclusion:*** The COVID-19 pneumonia epidemic has had a profound effect on consumer behavior worldwide. Under the current economic depression, the government should take adequate measures in order to respond to the new changes in consumer behavior and therefore promote economic growth. For example, the government should encourage the combination of online and offline business operation modes to break the boundaries of customer groups and supply chains, so that consumers can buy anytime and anywhere.

## 1. Introduction

Since the arrival of the COVID-19 pandemic in 2019, normal living and working conditions have been broken and this subsequently led to changes in consumer behaviors and, to a certain extent, living habits worldwide. In the face of these changes, and in order to better adapt to the developing needs of the modern consumer market, it is necessary to carry out a reasonable and effective analysis of consumer behavior in the context of the COVID-19 epidemic, and to put forward some practical suggestions for the modern market, thus contributing to the COVID-19 epidemic and laying a good foundation for sustainable economic development.

The theoretical definition of consumption is constantly developing and improving. In the field of early economic theory, consumption is defined as the process that people use to consume material that meets their own production and living needs. As one of the main participants in market economic activities, the academic circle has also conducted some research on the definition of the consumer. For example, Feng et al. (2004) have argued that two perspectives can influence consumer objectives. Consumer, in a narrow sense, refers to an individual who buys and uses all kinds of goods or services, whereas consumer in a broader sense refers to a consumer in a narrow sense. According to the definition of consumers, organizations introduce the purchase and use of goods or services [1]. The research summary of this paper is more from the narrow consumer perspective.

Traditional consumer behavior theory has been studied for half a century. From as early as the 1960s, some scholars have conducted relevant research on consumer behavior. Since then, the definition of consumer behavior in academia has been continuously developed and improved. The foreign scholar Wood (1981) believes that in a narrow sense, consumer behavior refers to a series of actions that people take to get what they need, such as the purchase of goods [2]. Engel (1986) believes that consumer behavior can be described as the various actions people take to acquire, use, and dispose of consumer goods and the decision-making process that precedes and determines these actions [3]. This definition emphasizes that consumer behavior is a whole, a process, and that acquisition or purchase is only one stage of this process. Schiffman and Kanuk (1987) believe that consumer behavior is all of the behaviors consumers show when they are looking for, buying, using, evaluating, and processing goods or services that they wish to acquire in order to meet their own needs [4]. Chen (2006) believes that consumer behavior is the basis for people to perform transactional functions in their lives and is easily affected by other factors, such as individual perception and environment [5]. The research on the definition of consumer behavior in the academic world is constantly being added to. From an economics perspective, the economics community believes that consumers are rational when making decisions, and they purchase goods or services based on the principle of maximizing benefits [6]. However, some scholars later pointed out that consumers are not wholly rational, and sometimes there are irrational buying behaviors. Their decision-making process is easily affected by many factors, such as personal cognition and personal emotions. Wang et al. (2012) used the term “utility” to measure consumer behavior, in which marginal utility theory and information asymmetry theory provided support for their views [7]. Marginal utility theory assumes that consumers are rational people and believes that consumers always hope to achieve maximum output with the smallest input to maximize total utility. The theory of information asymmetry assumes that the market is incomplete. The belief is that there is no symmetry in the information of the two parties in the market, and of the two it is the consumers who have less information, putting them at a disadvantage. Therefore, in the decision-making process of purchasing goods, consumers always want to collect as much product information as possible to make better decisions. In addition, many scholars have defined consumer behavior from other perspectives. For example, Lu (2017) proposed that consumer behavior is a decision-making process from a behavioral perspective. During this process, some content can be observed, such as the number of products purchased by consumers, and some content cannot be, such as the psychological activities of consumers when making decisions [8].

Consumer behavior theory has experienced radical changes and new situations, especially with the impact of COVID-19. With the development of the information age, consumer behavior is no longer a passive process but one that actively accepts new things. After the COVID-19 pneumonia epidemic outbreak, some scholars have also conducted some theoretical studies on consumer behavior. During the epidemic period, foreign scholars conducted very little research on consumer behavior definitions, and rather than significantly updating them, continued to use existing theoretical research. In contrast, Chinese studies on consumer behavior during the epidemic have made some supplements and improvements on the existing basis. The Chinese scholar Sun (2021) proposed that with the increase in the income of Chinese residents, under the social changes and rapid economic development, the consumption behavior of residents gradually changes, and these changes have promoted high-quality economic growth to a certain extent [9]. Li (2020) uses a stimulus-response model to explain consumer behavior and believes that stimulus is a key factor that affects consumer behavior. There are intermediate variables between stimulus and behavior, and more complex relationships exist between variables [10]. In addition, in the article, Li (2021) mentioned that consumer behavior research is how individuals, groups, and organizations choose, purchase, and use, in order to meet their own needs and desires [11]. For the study of consumer behavior, not only must one consider the important factors affecting consumer psychology and behavior, but one must also consider the analysis of various consumer psychological profiles and the behavior of different consumers. Jin (2021) pointed out that research on consumer behavior cannot be completely separated from the external background of the times, and more consideration should be given to changes in the social environment. Only by paying more attention to practical issues can we better promote the sound development of the discipline [12].

To sum up, COVID-19 has had a huge impact on people’s lifestyle and consumer behavior, and has also posed a great challenge to the traditional consumer behavior theories. Therefore, a study on the changes of consumer behavior theories since the outbreak of COVID-19 and its comparison with traditional consumer behavior theories will be of great value and, as such, this is the source of inspiration for this paper. This paper reviews the new changes in consumer behavior theory from four aspects: theoretical models of consumer behavior; consumption willingness; consumption patterns; and consumption objects. The hope is that the analysis of the new characteristics found in consumer behavior can provide a certain reference value and theoretical basis for the government to implement consumption policies and the consumption marketing of enterprises.

## 2. Materials and Methods

### 2.1. Search Process

The research topic of this paper is the latest development of consumer behavior theories under the impact of COVID-19. Firstly, the subject scope, period, and geographical boundary of the required literature were determined. Secondly, we found the necessary documents using familiar search tools and information sources according to its existing conditions. The literature was collected through CNKI, Wanfang, Google Scholar, Web of Science, and other platforms. The literature searches were conducted using keywords, journal names, institutions, etc. Keywords: COVID-19; consumer behavior; consumption theory; literature retrieval from February 1981 to October 2021. According to the specific selection criteria, we selected the literature that met the search requirements.

### 2.2. Inclusion Criteria, Extraction Process, and Quality Appraisal

**Figure 1:**
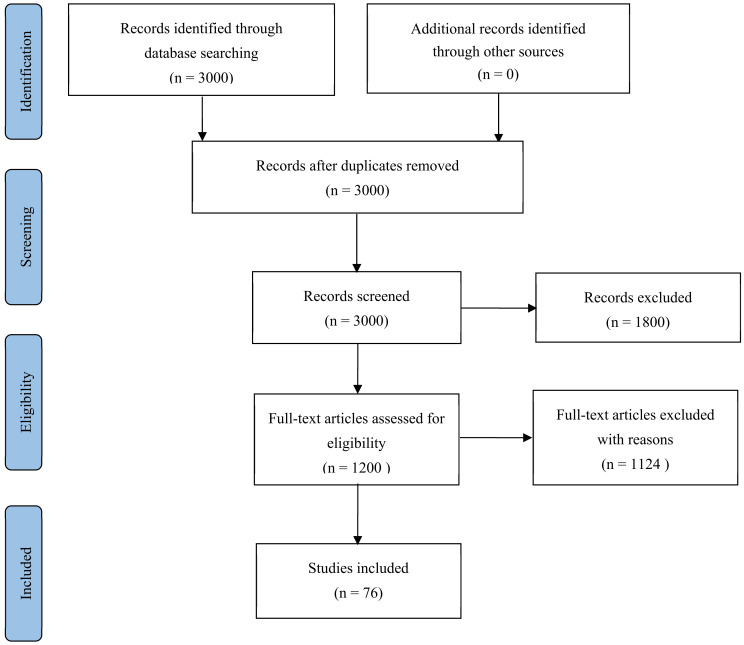
Data extraction process (PRISMA).

Within the literature on consumer behavior research, from as early as the 1960s, scholars have proposed that the time span is relatively long, and the geographical span is relatively large. After reading many documents, this article initially selected nearly 3000 related documents. However, considering the theoretical novelty of the literature, as well as the relevance and importance of the topic, the following 76 pieces of literature were selected according to the research requirements. The literature selected in this paper mainly focuses on the relevant research conducted in the last two decades. Of course, some old but theoretical classics of early research are kept as a supplement. Regarding the research on consumer behavior under the impact of the COVID-19 epidemic, the selected literature mainly focuses on the period from 2020 to 2021. The selected literature is closely related to the background of the times and is time-effective and convincing. Only publicly published and the most necessary documents are selected in this article, and the references are closely connected to the research topic and are true (as shown in Figure 1).

Our review methodology followed the checklist presented by PRISMA (the Project for Systematic Review and Meta-Analysis Preferred Reporting), which consists of a checklist and a flow diagram. Three reviewers appraised the studies for inclusion. The material extracted from the included publication included the study purpose, analysis tools, outcome measurements, and time frames. The data extraction process was made in Figure 1. Moreover, the JBI Critical Appraisal Checklist was used to evaluate the quality of the articles.

## 3. Results

### 3.1. Advances in Theoretical Models

#### 3.1.1. Early Research

The technology acceptance model is the main research model of consumer behavior theory. Based on the rational behavior theory, Davis et al. (1989) introduced a technology acceptance model, optimized the model accordingly, and then proposed a theoretical study of the technology acceptance model [13]. When the backend follows the technology acceptance model, innovations are made to a certain extent. Based on the original technology acceptance model, Yilmaz et al. (2011) supplemented the perceived service quality and information quality, and other influencing factors and used them to analyze online shopping behavior [14]. Angel Hern et al. (2011) added the factors of perceived compatibility and perceived usefulness to the technology acceptance model, and further analyzed the influencing factors of consumer behavior [15]. Domestic scholars also used the technology acceptance model when analyzing consumer behavior and adjusted and enriched it. Guo et al. (2018) added factors such as quality and price to the technology acceptance model, analyzed the influencing factors of consumer behavior on this basis, and found that shopping attitudes positively impact consumer intention [16]. When early scholars used the technology acceptance model to study consumer behavior, most of them focused on the factors influencing consumer behavior and optimized the previous models.

In addition to the technology acceptance model, other scholars have also put forward some theoretical research, a more typical theory of planned behavior. The foreign scholar Ajzen (2001) used the theory of planned behavior to analyze the individual decision-making process and showed that the theory of planned behavior could produce a certain degree of predictive and explanatory power on consumer behavior. The theory of planned behavior believes that the development of consumer behavior is a continuously strengthening process, which is the result of a combination of multiple factors [17]. Arvola, Verbekeb (2008) studied the consumption behavior of Italian, British and Finnish residents concerning organic food and confirmed that the theory of planned behavior has an effective predictive power on consumer intention [18]. Cook (2012) introduced the self-identity factor into the theory of planned behavior and used it to verify the degree of explanation and predictive power of the consumer behavior of genetically modified food [19]. Cheng and Tung (2014) added a moral trust factor to the theoretical model of planned behavior and verified that consumer environmental awareness affects green hotel consumption behavior [20]. Domestic scholars also have certain research on the theory of planned behavior. Liu (2008) verified that the theory of planned behavior could be used to explain and predict the green behavior of Chinese consumers [21]. Chen (2012) introduced personal factors into the theoretical model of planned behavior, which verified the explanation of herbal consumption willingness and consumer behavior by the theory of planned behavior [22]. When scholars use the theory of planned behavior to study consumer behavior, they do not just copy it but add some new factors into the theoretical model based on the original research.

#### 3.1.2. Latest Research: Shocks from COVID-19

In the early research on consumer behavior theory, the technology acceptance model and the planned behavior theory model appeared earlier and seemed more typical, however they were relatively outdated and did not contain novelty or innovation. During the COVID-19 pandemic, many scholars at home and abroad updated their theoretical research methods when researching consumer behavior theories. For example, during the epidemic, foreign scholars Omar NorAsiah et al. (2021) used behavioral inhibition system theory, resistance theory, and expectation theory to investigate psychological factors such as uncertainty, perception of severity, perception of scarcity, and anxiety to consumers panic buying and how these influenced behavior [23]. Koengkan Matheus (2021) used the moment quantile method to study consumer behavior and found that the overweight epidemic has increased the consumption of processed food from multinational food companies, fast food chains, and multinational supermarket chains [24]. During the COVID-19 period, foreign scholars have abandoned the old models and adopted relatively new research methods in the theoretical research of consumer behavior.

Of course, domestic scholars have also conducted some theoretical research. Wu (2021) studied the mechanism of the epidemic’s impact on the tourism industry in Guizhou Province based on the PSR theoretical model. The results showed that the epidemic affected residents’ intention to travel and consumption willingness, thereby affecting resident consumption in tourism [25]. Some scholars also studied the psychological state and emotions of residents. Zhang et al. (2021) used theoretical analysis, model construction, questionnaire surveys and other methods in the intersection of economics and psychology to analyze consumer behavior characteristics and reveal the main factors that affect consumer behavior regarding the buying of masks during the outbreak of the COVID-19 epidemic [26]. Sun et al. (2021) collected research data during the epidemic through a questionnaire survey and based this on a binary logistic regression model to explore the reasons for panic buying behavior in emergencies. The research results show that consumer education level and panic will significantly affect panic buying intentions [27]. Scholar research on consumer behavior theory during the epidemic is relatively new and time sensitive.

### 3.2. Consumption Willingness

#### 3.2.1. Early Research

Willingness is a concept in the category of psychology, which refers to the personal motivation in the conscious plan to make efforts to implement a behavior (Eagly, 1993) [28]. Ajzen (1991) mentioned in the theory of planned behavior that the intention to consume directly determines how consumers will adopt consumer behaviors and the size of the possibility of adopting specific consumer behaviors [29]. Newberry (2003) proposed that consumer behavior is directly related to consumption willingness, and consumption willingness is an important indicator when measuring whether consumers will have purchase behavior [30].

Regarding the study of consumer intention, early foreign scholars conducted many practical investigations. Some scholars tend to use the structural equation model to carry out research. Kim Minjung et al. (2013) surveyed smartphone users aged 20–30 and analyzed them using structural equation models. The results showed that perceived usefulness affects buying attitude, and buying attitude has a positive effect on purchase intention, so that perceived usefulness is, through purchase attitude, directly or indirectly having an impact on purchase intention [31]. Lee Jung Woo et al. (2016) collected data through questionnaire surveys and used AMOS 19.0 for confirmatory plant analysis, structural equation modeling, and multi-group analysis. This played an intermediary role and the recommendation information increased the consumer intention to buy [32]. Other scholars have also made some updates in their research methods. Kim T et al. (2017) asked 80 volunteers to participate in sensory tests on three smartphones and used one-way analysis of variance for posterior testing for Duncan’s test to study mobile shoppers’ intention to buy clothes [33]. Painter et al. (2018) selected US residents as the survey subjects in order to study whether US residents would be willing to pay for the Ebola vaccine during the 2014–2016 Ebola virus epidemic in West Africa [34]. Early foreign scholars tended to use sample surveys to collect data to study resident consumption willingness.

Domestic scholars have also researched consumer intention in the early stage, and most of them are theoretical. For example, from a theoretical point of view, Wang (2003) proposed that out of fear of death in the context of the epidemic, people began to converge blindly and follow the trend, and the consumer mentality of convergence has been strengthened, thereby changing their intention to consume [35]. Li et al. (2013) studied the influence mechanism of consumer identity on brand purchases. They proposed that consumer identity, positive emotions, and denial attitudes significantly impact brand choice intentions, and positive emotions and denial attitudes are between consumer identity and brand purchase intentions. Playing a significant intermediary role, brand self-consistency has a significant moderating effect between denial attitude and brand purchase intention [36]. In addition to analyzing the changes in consumer attitudes and consumer psychology, some scholars also analyzed the influence of the external environment on consumer intention. Li et al. (2018) believes that young people’s intention to consume online will be affected by the complexity of the consumption environment. Specifically, factors such as the image, word-of-mouth, brand, and consumer safety of e-commerce platforms will affect consumer satisfaction, affecting consumption willingness and consumption decision [37]. Contrary to foreign scholars, domestic scholars tend to analyze the impact of consumption environment, consumer attitude, and consumption psychology on consumption willingness, and there are many theoretical studies.

#### 3.2.2. Latest Research: Shocks from COVID-19

At present, foreign scholars have conducted a series of studies on consumption willingness in the context of the COVID-19 epidemic (Table 1). In 2016, Yogam et al. (2019) used a stratified two-stage cluster sampling method to sample 728 households in Serango, Malaysia, for interviews and used the conditional value method to estimate the intention to consume the hepatitis B vaccine and to understand Malaysian Serango adults during the epidemic and what factors affected their intention to consume the hepatitis B vaccine [38]. Some scholars have studied the intention to consume digital products and related media. Deng et al. (2020) analyzed the changes in consumer intention from different life scenarios such as life, work, study, and entertainment, summarized the development trend of digital products in the post-epidemic era, and put forward the main points of future digital product design [39]. Bendau (2020) studied related media consumption during COVID-19 and found that ordinary Germans’ anxiety, depression, and COVID-19-related fear symptoms affected the ordinary intention to consume related media [40]. There are also scholars who conduct research from the perspective of health tourism. Yin et al. (2021) used 306 online questionnaires, using COVID-19 risk information disclosure as the moderating variable and health awareness and risk perception as the intermediary variables, to investigate the impact of COVID-19 on online organic agricultural product consumption and rural health tourism consumption willingness [41]. Scholars from other countries besides China have conducted theoretical discussions on consumption willingness during the COVID-19 outbreak from different aspects.

Compared with other countries, Chinese scholars have conducted more detailed research on consumption willingness based on the background of the times in which we are currently living. During the period of COVID-19, research on consumption willingness mainly tends to analyze the impact of some intermediary pathways. From a practical point of view, Zhang et al. (2020) found through a questionnaire survey that consumer risk perception during the epidemic period hurts consumer intention through a chain-like intermediary path of negative emotion and safety motivation [42]. Wang et al. (2021) used the public perception and behavior survey data of major animal epidemics to explore the mechanism of the epidemic’s impact on consumption willingness using structural equation models. They found that gender, age, and education level will change consumption willingness in some ways [43]. Some scholars conducted comparative studies on consumption willingness before and after the epidemic. Sun et al. (2021) studied the consumption behavior of college students in Jiangning University Town and found that the consumption willingness of college students during the epidemic did not change much compared to before the epidemic, although there was some retaliatory consumption [44].

### 3.3. Consumption Patterns

#### 3.3.1. Early Research

Wang (2003) proposed that consumption patterns are different from the consumer behavior model and consumption culture. Consumption patterns are a structural concept, a structural or institutional arrangement of consumption by society [45]. To put it simply, the mode of consumption is the unity of a certain consumption tool and consumption system. The traditional way of consumption refers to the state of consumption before the emergence of new ways of consumption, and the most typical one is cash consumption. Cash consumption is only suitable for offline consumption, which has the characteristics of low efficiency and cumbersome links. With the rapid development of the Internet and the diversification of lifestyles, and the emergence of new payment methods such as Alipay, WeChat payment and credit card payment, traditional consumption methods have quietly changed and are gradually being replaced by these new consumption methods. In recent years, new online consumption methods have continued to emerge, and have made up for the shortcomings of offline consumption.

Judging from the existing relevant literature, there are fewer studies on offline consumption patterns, and they are older. Theoretical research on consumption patterns generally focuses on online consumption. For example, through online experiments, Li (2008) explored the influence of a web page background based on a priming effect had on consumer online shopping behavior, and the difference in online shopping behavior of consumers of different genders affected by web background. Online shopping behavior has an impact, and women’s online shopping behavior is more susceptible to the influence of web page background [46]. Some foreign scholars also discussed online shopping from the information aspect. Catherine Demangeot et al. (2010) believed that a large number of pages, products, and information capabilities are stored on the Internet, which increases the role and frequency of exploration in online shopping and proposed that it is important to redefine the exploration of online shopping [47]. Kwanho Kim (2013) researched that the abundance of product information provided by online shopping malls has a significant impact on consumer online buying behavior [48]. In addition, some scholars have investigated the online reputation and brand effect. Based on a questionnaire survey and analytic hierarchy process, Sun et al. (2014) researched that online store reputation and product cost-effectiveness are vital factors that affect consumer online buying behavior [49]. Suyanto Bagong et al. (2019) believe that young people are more susceptible to brand effects in the consumer group and therefore consume online [50]. The early studies of domestic and foreign scholars on online shopping tend to analyze the impact of webpage information, commodity information, and online shop reputation on consumer behavior.

#### 3.3.2. Latest Research: Shocks from COVID-19

Foreign scholars have conducted research and analysis on the online consumption patterns of residents during the epidemic period through relevant practical investigations (Table 2). Hanliang Fu et al. (2020) proposed that consumers rely on evaluating safety perception when purchasing goods online: the public judgment on the goods [51]. Jamunadevi C (2021) highlighted the transition of consumer behavior from physical stores to online shopping and believed that the COVID-19 virus has had an essential impact on the consumer buying behavior and will change future shopping habits [52]. Some foreign scholars analyzed the effect of COVID-19 on online shopping by using data collected from surveys. Pham Van Kien (2020) used the collected data of 427 Vietnamese online respondents to study and analyze the role of COVID-19 as a moderating variable between consumer perception of interest and their online shopping activities. The results show that COVID-19 encourages consumers to shop online [53]. Mouratidis Kostas et al. (2021) used the data of the Greek national survey to study the importance and frequency of participating in online activities before and during COVID-19 [54]. The outbreak of COVID-19 has encouraged residents to make online consumption, increased the frequency of online shopping, and changed consumption habits.

In contrast, domestic scholars analyze online consumption more in light of the current social background. Wu (2019) builds a research model based on the core factors of the technology acceptance model. The perceived usefulness of online shopping, ease of use, and attitudes toward e-commerce platforms all significantly impact consumer online shopping behavior [55]. In addition, during the epidemic period, Xie et al. (2020) found that most respondents are more inclined to use online shopping and contactless delivery [56]. Some scholars have discussed online and offline consumption theoretically. Tao and Zou (2020) pointed out that under the influence of the COVID-19 epidemic, the changes in consumer behavior have caused a huge impact on the offline consumption, although it has also brought some opportunities for the reform of the industry. For the healthy development of the Internet economy, the offline should be accelerated with the online integration process [57]. Zhu et al. (2021) analyzed the domestic economic development during the COVID-19 epidemic. They found that the epidemic hindered offline consumption, promoted online consumption, and suggested that the integrated development of the online and offline economies should be accelerated [58]. The COVID-19 epidemic has had a certain impact on consumption patterns, and domestic scholars advocate the combination of online and offline consumption to promote economic development.

### 3.4. Consumption Object

#### 3.4.1. Early Research

In the early domestic and international studies on consumer objects, there are a relatively large number of domestic studies. For example, from a theoretical point of view, Hua (2007) proposed that consumption is the driving force of economic growth, and all cultural products can be transformed into consumption objects [59]. From a practical point of view, Liu et al. (2012) conducted a questionnaire survey on college students from 3 colleges and universities in Chengdu. They analyzed the collected data to find out the law of college students’ travel consumption preferences and proposed optimization strategies for related tourism companies [60]. In addition to tourism consumption, some scholars have also researched leisure consumption objects. Li (2016) conducted a questionnaire survey on urban and rural residents in 10 cities, studied the leisure consumption objects of Chinese residents, analyzed the problems existing in the leisure consumption of Chinese residents, and made specific recommendations [61].

The above refer to the research dynamics under normal circumstances. When a major emergency occurs, the behavior of consumers will change as the external environment changes, so consumer objects will also vary accordingly. For example, in the context of emergencies, the accompanying consumer rushing behavior often occurs, and the consumption objects of residents also undergo a certain degree of change. Scholars at home and abroad have also conducted specific research on the snap-buying behavior during emergencies. The foreign scholars Chow et al. (2005) discovered that people were queueing up to buy gasoline after a hurricane damaged an oil production platform [62]. Hiroo Wada (2011) proposed that in the season of influenza virus infection, frequent hand washing behaviors will increase the consumption of disposable toilet paper [63]. Domestic scholar Duan et al. (2014) used the event research method to analyze the phenomenon of Banlangen panic buying caused by the H7N9 bird flu incident in my country in 2013 and studied the reasons for the abnormal income generated by such a panic buying storm [64]. Under abnormal circumstances, the consumption behavior of residents will change significantly, and the buying behavior of residents often occurs due to emergencies, and the consumption objects also change.

#### 3.4.2. Latest Research: Shocks from COVID-19

There are many and more detailed domestic and foreign studies on the consumption objects of residents during the epidemic (Table 3). Foreign scholar Duonamou Lucie (2020) studied the impact of consumer perception on consumer behavior and reported on meat and fish consumption behavior during the Ebola epidemic in West Africa and Guinea [65]. Many scholars have studied the consumption behavior of some teenagers during the COVID-19 pandemic [66]. Vasile G et al. (2021) learned the daily environmental-related activities of Romanian students during the COVID-19 family imprisonment and found that the consumption of water, plastic, and paper increased significantly during the epidemic [66]. The RogésJudit (2021) study found dangerous changes in the consumption of alcohol, tobacco, and marijuana caused by the COVID-19 lock-down in a group of educated adolescents in Central Catalonia in 2020 and analyzed the effects of individual factors and social factors. This showed the impact of risky consumption during the epidemic [67]. In addition, some scholars have researched the consumption of antimicrobial drugs. Al-Azzam Sayer (2021) studied the impact assessment of national antibacterial drug consumption during the epidemic. Certain antibiotics increased during the epidemic in relation to increased drug resistance [68]. According to the above study, the outbreak of COVID-19 has significantly increased the consumption of some daily necessities and certain antibiotics and also had a certain impact on risk consumption.

In addition, the epidemic has also had a huge impact on the tourism industry, constituting a dangerous external event, and has resulted in a tourism crisis. Based on actual data, Zhang (2021) analyzed the main energy supply and demand statistics before and during the epidemic and revealed the impact of COVID-19 on energy consumption in commercial tourism cities [69]. Fan et al. (2021), based on the concept and stage of the tourism crisis, put forward the problems faced by travel agencies and suggestions for promoting the high-quality development of travel agencies in response to the prevention and control process of the COVID-19 pneumonia epidemic [70]. On the contrary, the health consumption demand of residents has surged after the COVID-19 epidemic. Caso Daniela (2021) conducted a longitudinal study of food consumption during and after the first COVID-19 closure in Italy, and found that food consumption patterns before and after the closure have improved, providing interesting insights into the factors that promote healthy eating during future outbreaks. Theoretical contribution can be seen in [71]. In addition to healthy diet consumption, many aspects have fueled health awareness. After the COVID-19 pneumonia epidemic, Xu (2020) proposed that consumption consciousness showed a trend in health, space, effort-saving, and insurance consciousness [72]. Ni (2021) proposed that consumers’ attention to their health has risen to a new height in the post-epidemic era, and health consumption is gradually infiltrating more areas of life [73]. According to the above research, we have found that after the outbreak of COVID-19, health awareness has been significantly improved, and the demand for health consumption is also increasing day by day.

## 4. Discussion

COVID-19 has not only severely impacted consumer behavior, but also brought new realistic material to the development of consumer behavior theories. Therefore, the research on this issue not only has a certain frontier but also has important theoretical value. This paper tries to review the new changes of consumer behavior theory from four aspects: consumer behavior theoretical model; consumption intention theory; consumption mode theory; and consumption object theory. This research is very rare at present, and it has found some interesting phenomena.

By sorting out relevant research on consumer behavior theory, this paper finds that, even in the context of COVID-19, few studies on the definition of consumer behavior and no significant updates have been made, while Chinese scholars have made some supplements. Furthermore, some interesting conclusions are obtained. Firstly, early research mainly focused on the technology acceptance model and planned behavior theory in consumer behavior theory. In the context of COVID-19, theoretical models and research methods are more diverse, novel and time-sensitive. Secondly, regarding consumption willingness, early studies and the impact of COVID-19 mainly focused on the analysis of factors influencing consumption willingness. Thirdly, in terms of consumption patterns, both early studies and those under the impact of COVID-19 have focused on online consumption. However, in the context of COVID-19, scholars have proposed the focus should be on the integration of online and offline development. Finally, in terms of consumption objects, compared with previous studies, in the context of COVID-19 and some panic buying behaviors, scholars also believe that the demand for healthy consumption has increased. All these have greatly affected people’s economic welfare. As Akram (2021) believes, online consumption and digitalization during COVID-19 not only have a great impact on customers’ well-being, but also pose great challenges and development opportunities to the retail industry [74].

The COVID-19 pandemic has had a profound impact on consumer behavior. In the current economic downturn, the government should take adequate measures to cope with the new changes in consumer behavior to promote economic growth. Firstly, in terms of consumption willingness, the government should encourage the continuous emergence of new products, new services, and new forms of business to stimulate consumer consumption potential, enhance consumer confidence [75] and thus increase their consumption willingness. Secondly, in terms of consumption patterns, the government should greatly improve the social commerce environment for online business [76]. For example, the business operation mode of full resource sharing between online and offline can be actively utilized to break the online and offline boundaries, customer group boundaries, supply chain boundaries, platform boundaries and geographical boundaries, so that consumers can truly shop anytime and anywhere. Thirdly, in terms of consumption objects and due to the impact of the epidemic, awareness of healthy consumption continues to increase. Therefore, the government should actively expand domestic health consumer demand, meet consumer demand, and provide more satisfactory products and services. What should be noted is that, due to the content of the previous literature and the author’s level, this paper leaves plenty of room for improvement. For example, the theoretical model of consumer behavior and the specific types of consumer behavior are not researched thoroughly enough, so we hope to improve upon these aspects in future research.

## Figures and Tables

**Table 1:** Research on consumer intention.

Author	Research Object	Analysis Method	Research Results
Kim M (2013)	Smartphone users aged 20–30	Structural equation model	Perceived usefulness directly or indirectly affects purchase intention through purchase attitude
Lee J (2016)	Consumers at large	Confirmatory plant analysis, structural equation modeling	If the recommendation information of acquaintances acts as an intermediary, the recommendation information will increase the consumer’s intention to buy
Kim T (2017)	80 volunteers	One-way analysis of variance, Duncan test	The phone does not represent the “real” fabric color
Painter J (2018)	U.S. residents	Investigation and analysis	The intention of US residents to pay for ebola vaccine increased significantly during the Ebola epidemic in West Africa
Yogam (2019)	728 households in Serango	Malaysia Conditional Value Method	To explore and analyze the influencing factors of Malaysian Serango adults’ intention to consume hepatitis B vaccine
Zhang (2020)	Consumers at large	Questionnaire analysis	It is found that consumer risk perception during the epidemic period has a negative impact on consumer intention through the chain-like intermediary path of negative emotions and safety motives
Yin J (2021)	Consumers at large	Questionnaire analysis	Investigate the impact of COVID-19 on online organic agricultural product consumption and rural health tourism Consumption willingness
Sun (2021)	College students in Jiangning University Town	Investigation and analysis	It was found that during the epidemic period, college students’ intention to spend has not changed much compared with before, but there is some retaliatory consumption
Wang (2021)	The public residents	Structural equation model	gender, age, education level, etc. will change their intention to consume in some ways

**Table 2:** Research on consumption patterns.

Author	Research Object	Analysis Method	Research Results
Li (2008)	Consumers at large	Online experiment	Web page background has an impact on consumers’ online shopping behavior, and women’s online shopping behavior is more susceptible to the influence of web page background
Sun (2014)	Online consumer	Questionnaire and analytic hierarchy process	Online store reputation and product cost-effectiveness are key factors that affect consumers’ online buying behavior
Wu (2019)	Consumers at large	Technology acceptance model	Perceived usefulness, ease of use, and attitudes toward e-commerce platforms in online shopping all have a significant impact on consumers’ online shopping behavior
Pham Van Kien (2020)	427 online Vietnamese respondents	Survey analysis method	COVID-19 encourages consumers to shop online
Xie (2020)	Consumers at large	Questionnaire	Most survey respondents are more inclined to online shopping and contactless delivery
Jamunadevi C (2021)	Online consumers during the COVID-19 epidemic	Model analysis	The COVID-19 virus has an important impact on consumers’ buying behavior, and it will change consumers’ future shopping habits
Mouratidis Kostas (2021)	Consumers across Greece	Investigation and analysis	The importance and frequency of online shopping has increased significantly during COVID-19
Zhu (2021)	Economic development during the domestic epidemic	The theoretical analysis	The epidemic hinders offline consumption and promotes online consumption

**Table 3:** Main research on consumer objects.

Author	Research Object	Analysis Method	Research Results
Liu (2012)	College students from 3 universities in Chengdu	Questionnaire analysis	Exploring the Law of College Students’ Travel Consumption Preference
Duan (2014)	Banlangen objects to buy	Event research	Analyzed the reasons for the abnormal returns caused by the panic buying of Banlangen
Li (2016)	Urban and rural residents in 10 cities	Questionnaire analysis	Analyze the problems existing in the leisure consumption of Chinese residents
RogésJudit (2021)	The educated youth group in Central Catalonia	The empirical analysis	Analyzed the influence of individual factors and social factors on risk consumption during the epidemic
Zhang (2021)	Energy supply and demand before and after the epidemic	Statistical analysis	Reveals the impact of COVID-19 on energy consumption in commercial tourism cities
GherheșVasile (2021)	Romanian students	Survey analysis method	During the epidemic, water, plastic and paper consumption increased significantly
Al-Azzam Sayer (2021)	National Antibacterial Drugs	Empirical evaluation analysis	Increased use of certain antibiotics during a pandemic related to increased resistance
